# Volumetric capnography and return of spontaneous circulation in an experimental model of pediatric asphyxial cardiac arrest

**DOI:** 10.1038/s41598-023-37827-1

**Published:** 2023-07-28

**Authors:** Sara de la Mata Navazo, Gema Manrique, Sarah Nicole Fernández, Gema Pérez, Laura Butragueño‑Laiseca, Miriam García, María Slöcker, Rafael González, Laura Herrera, Santiago Mencía, Jimena del Castillo, María José Solana, Débora Sanz, Raquel Cieza, Jorge López, Alicia Rodríguez Martínez, María José Santiago, Javier Urbano, Jesús López‑Herce

**Affiliations:** 1 Pediatric Intensive Care Department, Gregorio Marañón University Hospital, Dr Castelo 47, 28009 Madrid, Spain; 2 Health Research Institute of the Gregorio Marañón Hospital, Madrid, Spain; 3 Primary Care Interventions to Prevent Maternal and Child Chronic Diseases of Perinatal and Development Origin Network (RICORS) RD21/0012/0011, Carlos III Health Institute, Madrid, Spain; 4 Maternal and Child Public Health Department, School of Medicine, Complutense University of Madrid, Madrid, Spain

**Keywords:** Cardiology, Medical research

## Abstract

A secondary analysis of a randomized study was performed to study the relationship between volumetric capnography (VCAP) and arterial CO_2_ partial pressure (PCO_2_) during cardiopulmonary resuscitation (CPR) and to analyze the ability of these parameters to predict the return of spontaneous circulation (ROSC) in a pediatric animal model of asphyxial cardiac arrest (CA). Asphyxial CA was induced by sedation, muscle relaxation and extubation. CPR was started 2 min after CA occurred. Airway management was performed with early endotracheal intubation or bag-mask ventilation, according to randomization group. CPR was continued until ROSC or 24 min of resuscitation. End-tidal carbon dioxide (EtCO_2_), CO_2_ production (VCO_2_), and EtCO_2_/VCO_2_/kg ratio were continuously recorded. Seventy-nine piglets were included, 26 (32.9%) of whom achieved ROSC. EtCO_2_ was the best predictor of ROSC (AUC 0.72, p < 0.01 and optimal cutoff point of 21.6 mmHg). No statistical differences were obtained regarding VCO_2_, VCO_2_/kg and EtCO_2_/VCO_2_/kg ratios. VCO_2_ and VCO_2_/kg showed an inverse correlation with PCO_2_, with a higher correlation coefficient as resuscitation progressed. EtCO_2_ also had an inverse correlation with PCO_2_ from minute 18 to 24 of resuscitation. Our findings suggest that EtCO_2_ is the best VCAP-derived parameter for predicting ROSC. EtCO_2_ and VCO_2_ showed an inverse correlation with PCO_2_. Therefore, these parameters are not adequate to measure ventilation during CPR.

## Introduction

Capnography is a useful monitoring technique in cardiopulmonary resuscitation (CPR)^1^. It has multiple applications that include verifying adequate endotracheal tube position and assessing quality of chest compressions in adult CPR^2,3^. Moreover, a retrospective study that analyses 426 out-of-hospital cardiac arrest cases suggest an association between capnographic values and return of spontaneous circulation (ROSC)^4^.

Regarding pediatric CPR, there is insufficient evidence supporting the use of end-tidal carbon dioxide (EtCO_2_) as a as a marker of quality or as a prognostic marker during CPR. Therefore, the European Resuscitation Council guidelines^5^ advocate its use to monitor ETT position and state that it can help to rapidly detect ROSC, but do not recommend it as a marker of CPR quality, while the American Heart Association Guidelines^6^ state that EtCO_2_ monitoring may be considered to assess the quality of chest compressions^7^.

Time-based capnography, widely accessible, provides valuable information such as end-tidal carbon dioxide (EtCO_2_) values and capnography wave analysis. Other techniques as volumetric capnography (VCAP) offer a representation of CO_2_ production, transport and elimination, that could be useful during CPR, although they might not be always available in a CPR setting^8^. However, there are no published studies regarding the relationship between VCAP-derived parameters and real ventilation status (based on PCO_2_) in pediatric animal models of cardiac arrest (CA).

We conducted a secondary analysis of a previously published experimental study using a pediatric animal model of asphyxial cardiac arrest^9^, to analyze the relationship between VCAP-derived parameters and arterial blood CO_2_ partial pressure (PCO_2_) and their ability to predict ROSC.

## Methods

This study is a secondary analysis of the data obtained in a randomized prospective experimental study, performed in the Experimental Medicine and Surgery Department of a third level hospital in Madrid, Spain. The experimental protocol was approved by the Local Ethics Committee in Animal Research and authorized by the Autonomous Community of Madrid (reference number PROEX 096/19). The study was developed in compliance with the ARRIVE guidelines, and all methods were carried out in accordance with guidelines and regulations. The study protocol has already been thoroughly described in a previously published article^9^.

### Animal preparation and monitoring

Seventy-nine 3-month-old miniature pigs, weighing 9–12 kg, were included.

Arterial, peripheral and central venous lines were inserted and connected to a PiCCO system for hemodynamic monitoring (heart rate, arterial blood pressure cardiac index and temperature). ECG and pulse oximetry were monitored continuously. Cerebral (ScO2) and splanchnic (SsO2) oxygen saturations were monitored by near-infrared spectroscopy (NIRS) (INVOS Cerebral Oximeter monitor, Somanetics, Troy, Michigan, USA). Respiratory parameters were continuously monitored using a sensor placed at the Y piece and connected to a Respironics NM3 monitor (Philips Healthcare, Markham, ON, Canada). Maintenance fluids containing glucose and saline were infused. Animal temperature was kept between 37 and 39 °C. Chest compressions were performed guiding depth and rate with a defibrillator monitor (Zoll Z series).

### Experimental protocol

After instrumentation, sedation and an initial stabilization period, an asphyxial CA was induced by extubating animals after administering an intravenous dose of atracurium. CA was defined as a mean arterial pressure under 25 mmHg. Advanced CPR was started 2 min after diagnosing CA.

Animals were randomized in five different groups according to airway management (early intubation-ETI or bag-mask ventilation-BMV) and delivery of ventilation during CPR: with real-time tidal volume feedback (TVF) of 7 or 10 ml/kg, or without feedback, depending on chest expansion (standard ventilation—SV). Resuscitation groups are shown in Table 1. Resuscitation was continued until ROSC or up to a maximum of 24 min.

**Table 1:** Randomization groups during CPR.

Group number	Airway management	Delivery of ventilation	N	ROSC
Group 1	ETI	TVF—eTV 10 ml/kg	17	7 (41.2%)
Group 2	ETI	TVF—eTV 7 ml/kg	15	6 (40%)
Group 3	ETI	Standard ventilation	15	6 (40%)
Group 4	BMV	TVF—eTV 10 ml/kg	17	4 (23.5%)
Group 5	BMV	Standard ventilation	15	3 (20%)

*ETI* early tracheal intubation, *BMV* bag-mask ventilation, *TVF* tidal volume feedback, *eTV* exhaled tidal volume, *ROSC* return of spontaneous circulation.

### Study variables

Clinical and monitoring parameters were collected at baseline, 5 min after extubation, before the start of CPR and every 3 min during resuscitation. Arterial blood gases were withdrawn at baseline and after every 3 min of CPR. Each ventilation was recorded, and respiratory parameters were registered at baseline and every 3 min during CPR.

### Statistical analysis

The SPSS statistical package, version 25.0 (SPSS Inc, Chicago, USA) was used for statistical analysis. Continuous variables are expressed as means with standard deviation and categorical variables as percentages. The correlation between continuous variables was calculated with Pearson's correlation coefficient. To assess the sensitivity and specificity of the different variables to detect the return of spontaneous circulation, a ROC (receiver operating characteristic) curve was performed. The area under the curve and the Youden index were calculated. p values less than 0.05 were considered significant.

A sample size calculation was performed accepting an alpha risk of 0.05 and a beta risk of 0.2. ROSC was considered the main effect, with an estimated incidence of 30% based on previous experiences^10–12^ with the same animal model. An effect size of 25% on the usual incidence of ROSC was considered significant. A drop-out rate of 10% was estimated. Therefore, 15 animals per group were required, with a total number of 75 animals.

## Results

Seventy-nine piglets weighting 11.3 ± 1.2 kg were included in the study, with a ROSC rate of 32.9% (26 animals). ROSC rate according to randomization group is shown in Table 1.

**Figure 1:**
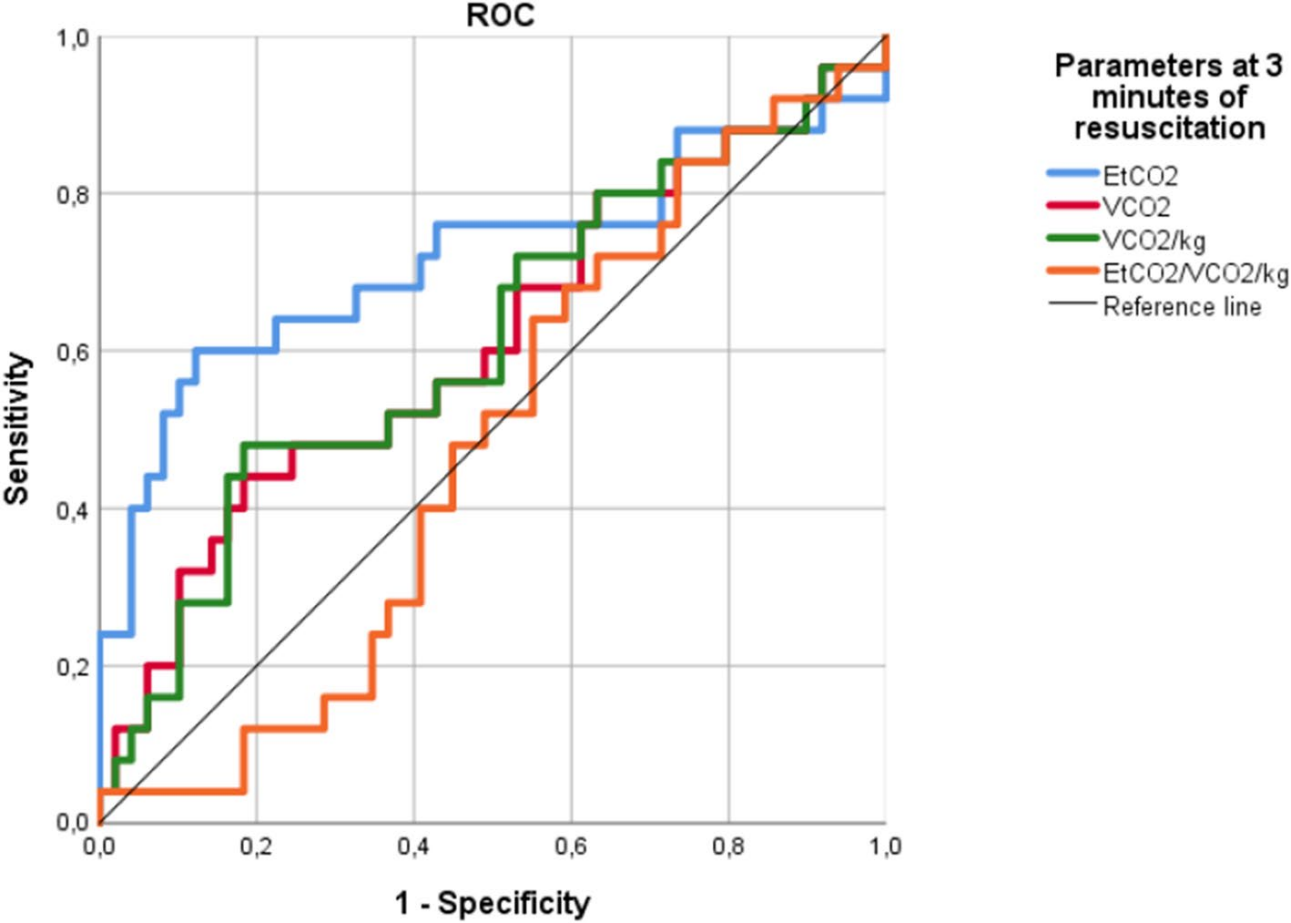
ROC curve showing performance of VCAP-derived parameters in predicting ROSC. *EtCO*_*2*_ end-tidal carbon dioxide, *VCO*_*2*_ CO_2_ production.

A receiver operator characteristic (ROC) curve that included EtCO_2_, VCO_2_, VCO_2_/kg and ratio EtCO_2_/VCO_2_/kg was performed, showing that EtCO_2_ at 3 min of resuscitation was the only predictor of ROSC, with an AUC of 0.71 (Table 2, Fig. 1). The optimal cut-off point for predicting ROSC was an EtCO_2_ value of 21.58 mmHg, with a sensitivity of 56% and a specificity of 89%. Sensitivity, specificity and Youden’s index for predicting ROSC of EtCO_2_ values of 10, 15, 20 and 25 mmHg are shown in Table 3.

**Table 2:** Area under the curve (AUC) of VCAP-derived variables obtained in ROC curve.

Variables	AUC	Signification
EtCO_2_ CPR 3	**0.72**	** < 0.01**
VCO_2_ CPR 3	0.61	0.12
VCO_2_/kg CPR 3	0.61	0.12
EtCO_2_/VCO_2_/kg CPR 3	0.48	0.79

**Table 3:** Sensitivity and specificity of EtCO_2_ values for predicting ROSC.

EtCO_2_ (mmHg)	Sensitivity	Specificity	Youden’s index
10	0.88	0.26	0.14
15	0.76	0.55	0.31
20	0.60	0.82	0.42
**21.58**	**0.56**	**0.90**	**0.46**
25	0.44	0.92	0.36

Significant values are in [bold].

Animals that achieved ROSC had higher median EtCO_2_ levels (30.1 vs 11.9 mmHg) than those who did not, regardless of airway management (intubation or bag-mask ventilation) after applying a linear multiple regression model (p < 0.01).

Linear correlation was performed to study the relationship between PCO_2_ and EtCO_2_, VCO_2_, VCO_2_/kg and ratio EtCO_2_/VCO_2_/kg (Table 4). A negative correlation was found between EtCO_2_ and PCO2 from resuscitation minutes 18 to 24. VCO_2_ and VCO_2_/kg had a statistically significant negative correlation with PCO_2_ during resuscitation, with more negative correlation as resuscitation time increased. Regarding EtCO_2_/VCO_2_/kg ratio, it was only significantly correlated with PCO_2_ at resuscitation minute 3.

**Table 4:** Pearson’s correlation between VCAP-derived parameters and partial arterial CO_2_ (PCO_2_) in different time points of resuscitation.

PCO_2_		EtCO_2_	VCO_2_	VCO_2_/kg	EtCO_2_/VCO_2_/kg
PCO_2_ 3' CPR	r	− 0.17	− 0.37	− 0.37	0.43
	p	0.15	< 0.01	< 0.01	< 0.01
PCO_2_ 6' CPR	r	0.21	− 0.29	− 0.30	0.16
	p	0.15	0.04	0.03	0.27
PCO_2_ 9' CPR	r	− 0.15	− 0.27	− 0.29	0.06
	p	0.31	0.05	0.04	0.70
PCO_2_ 12' CPR	r	− 0.25	− 0.58	− 0.58	0.15
	p	0.09	< 0.01	< 0.01	0.32
PCO_2_ 18' CPR	r	− 0.38	− 0.62	− 0.61	0.07
	p	< 0.01	< 0.01	< 0.01	0.64
PCO_2_ 21' CPR	r	− 0.44	− 0.57	− 0.55	0.03
	p	< 0.01	< 0.01	< 0.01	0.86
PCO_2_ 24' CPR	r	− 0.46	− 0.66	− 0.65	0.20
	p	< 0.01	< 0.01	< 0.01	0.18

## Discussion

This study is, as far as we know, the first experimental animal study that analyses the relationship between VCAP-derived parameters, survival, and ventilation status during pediatric cardiopulmonary resuscitation.

Our results show that EtCO_2_ is the best capnometric parameter to predict ROSC, which has already been demonstrated in previous publications^4,13–18^. We found that animals that achieved ROSC had higher median EtCO_2_ levels than non-survivors and we obtained an optimal EtCO_2_ cutoff point of 21.58 mmHg to predict ROSC.

A large cohort study that analyzed data of 143 pediatric cardiac arrest events^18^ reported a significant difference in median EtCO_2_ between events that achieved ROSC and those that did not. However, when data were stratified based on patient age, this relationship was only significant in adolescents, without differences in EtCO_2_ levels in children and infants that achieved ROSC and those who did not survive.

Several authors have analyzed EtCO_2_ cutoff points for predicting ROSC: Sorcher et al.^18^ used a cutoff point of 20 mmHg. Chalak et al.^14^ established an EtCO_2_ cutoff point of 14 mmHg in an experimental model of neonatal asphyxial CA, while Stine et al.^15^ reviewed CA episodes in patients less than 6 months hospitalized in an intensive care unit, setting an EtCO_2_ cutoff point of 17–18 mmHg. Both studies defined ROSC as the achievement of a heart rate higher than 60 beats per minute. Another study of in-hospital CA in adults set an EtCO_2_ cutoff point of 25.5 mmHg for predicting sustained ROSC^16^.

Given these differences between studies and the number of influencing factors (hyperventilation, drug administration, etc.)^1^, it is specially challenging to stablish an absolute EtCO_2_ cutoff point for predicting ROSC. Therefore, a case–control study of adult out-of-hospital CA suggested that CO_2_ trends were more useful to predict ROSC, as survivors had more positive trends than non-survivors^19^.

Some authors suggested that VCO_2_ and VCO_2_/kg were also good predictors of ROSC^17,20,21^, although these parameters did not demonstrate utility for this purpose in our study. Most of these studies induced a ventricular-fibrillation cardiac arrest. Nevertheless, cardiac arrest of sudden cardiac origin (such as ventricular fibrillation) is not associated with high CO_2_ levels at the beginning of CPR, as in CA of respiratory origin^22–24^. This fact could explain the different performance of VCO_2_ and VCO_2_/kg in our study. However, another experimental study of asphyxial cardiac arrest in neonatal piglets found that both EtCO_2_ and VCO_2_ during CPR were higher in survivors^25^.

Regarding the relationship between EtCO_2_ and PCO_2_, we found an inverse correlation from minute 18 to minute 24 of CPR. It would be expected that changes in PCO_2_ would be associated with parallel changes in EtCO_2_. Nevertheless, EtCO_2_ is greatly affected by pulmonary blood flow (as with decreased cardiac output), which can explain the negative correlation.

These findings were consistent with a previous study showing a progressive increase of PCO_2_ and decrease of EtCO_2_ throughout CPR^14^. Another study in out of hospital CA in adult patients, however, showed moderate correlation between EtCO_2_ and PCO_2_ during reanimation. Nevertheless, blood samples in that study were obtained at any point of CPR when an arterial line was obtained, regardless of the duration of CA or resuscitation^26^.

Zhang et al. found that EtCO_2_ was a useful parameter to predict ROSC, and that VCO_2_/kg ratio had similar capacity to predict ROSC in an experimental porcine cardiac arrest model^20^. This group also analyzed the relationship between EtCO_2_/VCO_2_/kg and ventilation during CPR in an experimental study with adult pigs, finding that this ratio showed good performance in discriminating hyperventilation from non-hyperventilation^13^.

Regarding VCO_2_ and VCO_2_/kg, our results show an inverse correlation of both parameters with PCO_2_, which became more significant as resuscitation progressed in time. VCO_2_ values are determined by tidal volume and EtCO_2_. Therefore, if tidal volume remains stable, VCO_2_ will be affected by the same factors that affect EtCO_2_: the decrease of cardiac output and pulmonary perfusion during CPR modifies ventilation/perfusion ratio, reducing expired CO_2_ fraction, and consequently, VCO_2_.

As for EtCO_2_/VCO_2_/kg ratio, we only found correlation with PCO_2_ at minute 3 of resuscitation. Zhang et al.^20^ reported that this parameter was a good predictor of hyperventilation, although PCO_2_ was not measured during resuscitation.

VCO_2_ measurement requires the use of a specific volumetric capnograph. Such capnographs are not widely available and, according to our results, this parameter does not accurately reflect ventilation during CPR. In the light of these findings, VCAP parameters do not seem to offer any additional benefits than regular capnography during CPR. However, these findings should be validated with specific pediatric clinical studies.

Our study has several limitations. Although we used a validated pediatric animal model for this purpose, the results from animal experiments cannot be directly extrapolated to children. Besides, the variability of VCAP measurement when using an endotracheal tube or a face mask could interfere with the results.

## Conclusions

In an experimental model of pediatric asphyxial cardiac arrest, EtCO_2_ was the only VCAP-derived parameter for predicting ROSC. VCO_2_ and VCO_2_/kg and EtCO_2_ had an inverse correlation with PCO_2_ during CPR. Therefore, they are not suitable for assessing PCO_2_ during resuscitation. These findings highlight the importance of measuring arterial CO_2_ partial pressure during CPR.

## Data Availability

The datasets used and/or analyzed during the current study are available from the corresponding author upon reasonable request.
